# Oseltamivir-Associated Angioedema in a Patient Receiving Angiotensin-Converting Enzyme Inhibitor Therapy for Influenza A: A Case Report

**DOI:** 10.7759/cureus.115714

**Published:** 2026-09-03

**Authors:** Opemipo A Dosumu

**Affiliations:** 1 Internal Medicine, University of Maryland Global Campus, College Park, USA

**Keywords:** ace-i, drug-induced angioedema, facial swelling, influenza virus type a, oseltamivir

## Abstract

Influenza remains a major cause of morbidity and mortality worldwide, particularly among elderly patients with comorbidities. Oseltamivir (Tamiflu), a neuraminidase inhibitor, is the most widely prescribed antiviral therapy for influenza A and B. Although oseltamivir is associated with increased nausea and vomiting, it is generally well tolerated. On rare occasions, oseltamivir has been associated with rare hypersensitivity reactions, including angioedema. Angiotensin-converting enzyme (ACE) inhibitors are also well known to cause angioedema, sometimes years after therapy initiation. We present the case of a 73-year-old man with influenza A who developed acute lip swelling after starting oseltamivir while on long-term lisinopril therapy. Oseltamivir was discontinued, and the swelling subsided. This raised important diagnostic and therapeutic considerations in differentiating viral illness, drug reaction, and medication-induced angioedema.

## Introduction

Seasonal influenza A virus infection remains a significant global health concern. Influenza A viruses were responsible for an average of 12,000 to 52,000 deaths annually between 2010 and 2020 in the United States [1]. Seasonal influenza results in approximately one billion cases annually worldwide, including three to five million cases of severe illness and up to 650,000 respiratory deaths [2]. Elderly individuals and those with chronic comorbidities remain at highest risk of complications. Antiviral therapy, particularly neuraminidase inhibitors, plays a critical role in reducing disease severity and preventing complications.

Since its introduction, oseltamivir has been widely used for both prophylaxis and treatment of influenza. While its most common adverse effects are gastrointestinal, including nausea, vomiting, and abdominal discomfort, less frequent but clinically significant reactions have been reported [3]. These include neuropsychiatric effects, hepatic toxicity, and hypersensitivity reactions ranging from mild rash to anaphylaxis [4]. Oseltamivir has also been associated with rare cases of tongue and lip swelling consistent with hypersensitivity reactions [4]. Oseltamivir, marketed as Tamiflu in the United States, remains one of the most widely prescribed antiviral therapies for influenza A and B due to its neuraminidase inhibition activity [5].

Hypersensitivity reactions, including angioedema, are rare but potentially life-threatening. Angioedema has also been strongly associated with angiotensin-converting enzyme (ACE) inhibitors, which are widely prescribed for hypertension and cardiovascular protection [6]. Importantly, ACE inhibitor-induced angioedema may occur even after many years of stable therapy [6]. When patients present with influenza infection, multiple potential triggers for facial swelling - including viral infection itself, oseltamivir therapy, and ACE inhibitor exposure - must be carefully considered.

This case highlights the diagnostic challenges and clinical implications of angioedema in a patient with influenza A who was receiving both oseltamivir and lisinopril.

## Case presentation

A 73-year-old male presented to the emergency department (ED) on February 5, 2024, with altered mental status. His past medical history included transient ischemic attack, pulmonary embolism, hypertension, hyperlipidemia, and benign prostatic hyperplasia. According to his nephew, the family had been unable to reach him for several days. Upon visiting, the nephew noted that the patient appeared weak, somnolent, and lethargic. The patient reported cough, chills, and generalized weakness but denied chest pain, abdominal pain, shortness of breath, or falls. Initial evaluations are presented in Tables 1-2.

**Table 1:** Patient's Vital Signs bpm: beats per minute; °F: degrees Fahrenheit

Parameter	Patient Value	Normal Reference Range (Adults)	Interpretation
Blood Pressure (BP)	124/84 mmHg	90-120/60-80 mmHg	Mildly elevated diastolic BP
Heart Rate (HR)	69 bpm	60-100 bpm	Normal
Temperature (°F)	98.7°F (37.1°C)	97-99°F (36.1-37.2°C)	Normal (afebrile)
Respiratory Rate (RR)	16 breaths/min	12-20 breaths/min	Normal
Oxygen Saturation (SpO_2_)	95% (room air)	≥95% on room air	Lower limit of normal

**Table 2:** Patient's Lab Results Bold values indicate abnormal results.

Laboratory Test	Patient Value	Normal Reference Range	Interpretation
Sodium (Na⁺)	143 mmol/L	135-145 mmol/L	Normal
Potassium (K⁺)	3.2 mmol/L	3.5-5.0 mmol/L	Low (hypokalemia)
Chloride (Cl⁻)	108 mmol/L	98-106 mmol/L	Mildly elevated (hyperchloremia)
Bicarbonate (HCO₃⁻)	24 mmol/L	22-29 mmol/L	Normal
Blood Urea Nitrogen (BUN)	25 mg/dL	7-20 mg/dL	Elevated
Glucose	143 mg/dL	<140 mg/dL (random)	Elevated

On physical examination, the patient was an elderly male who was somnolent and lethargic, with diminished bilateral lung sounds. Mild upper-lip edema was noted later during his ED stay. Cardiovascular and abdominal examinations were unremarkable.

A rapid molecular polymerase chain reaction (PCR) test for influenza A and B, SARS-CoV-2, and respiratory syncytial virus (RSV) was performed approximately one hour after arrival. The test was positive for influenza A. While in the ED, his home medications were continued, including lisinopril/hydrochlorothiazide 20/25 mg. The patient had been taking lisinopril/hydrochlorothiazide 20/25 mg for six years.

Following the positive influenza A result, the patient was started on oseltamivir 75 mg. Approximately one hour after taking oseltamivir, the patient went to the restroom and returned with sudden swelling of his upper lip. He remained hemodynamically stable, without tongue swelling, stridor, or respiratory distress. The upper-lip swelling persisted for approximately one hour. Oseltamivir was discontinued at that point. The swelling gradually began to improve over the following two hours, and by the time the hospitalist team evaluated the patient, it had significantly improved.

Given the close temporal relationship between oseltamivir administration and the onset of upper-lip swelling, oseltamivir was considered a possible trigger. However, the patient's long-term lisinopril therapy represented an important confounding factor, and ACE inhibitor-associated angioedema could not be excluded. The hospitalist team administered a single dose of intravenous diphenhydramine. The patient also received intravenous fluids during his hospitalization. The mild laboratory abnormalities noted on admission, including hypokalemia, mild hyperchloremia, elevated blood urea nitrogen (BUN), and mild hyperglycemia, had no documented specific treatment directed toward them. The patient was observed for 24 hours, during which the lip swelling resolved.

The patient was discharged on the morning of hospital day 3. He was scheduled for follow-up with his primary care physician four days after discharge. At follow-up, there was no reported recurrence of the lip swelling. Information regarding any subsequent changes to his antihypertensive regimen was not available to the treating hospitalist team.

## Discussion

This case highlights the challenge of identifying the cause of acute lip swelling in a patient with influenza A who was receiving several medications and had clinical factors associated with angioedema. The main possibilities included oseltamivir-induced hypersensitivity, ACE inhibitor-induced angioedema related to chronic lisinopril therapy, and inflammation associated with the viral infection itself. Because these conditions can present similarly, determining the exact cause can be difficult.

Oseltamivir is widely used to treat influenza because it is generally safe and effective [3,5]. Most adverse effects are mild and involve the gastrointestinal tract, while hypersensitivity reactions are uncommon [4,7]. Kalsi et al. [4] described tongue swelling shortly after oseltamivir administration, suggesting a possible hypersensitivity reaction. Peace et al. [7] also reported suspected oseltamivir-induced angioedema in a patient with chronic renal failure. Our patient had a similar temporal relationship between oseltamivir administration and the onset of upper-lip swelling. However, unlike these previous reports, our patient was also receiving long-term lisinopril therapy, making the diagnosis less straightforward.

ACE inhibitor-induced angioedema was an important alternative consideration. This adverse effect is well recognized and may occur even after years of treatment without previous symptoms [6]. Although the temporal relationship between oseltamivir administration and symptom onset supports oseltamivir as a possible trigger, the patient's six-year exposure to lisinopril means that ACE inhibitor-associated angioedema cannot be excluded. Influenza infection itself may also have contributed by increasing the inflammatory response. Therefore, more than one factor may have contributed to this patient's presentation.

Our case also differs from the report by Scott et al. [8], who described severe tongue swelling in a patient with influenza A (H1N1) requiring mechanical ventilation. In contrast, our patient had isolated upper-lip swelling without airway compromise and improved after discontinuation of the suspected offending medication and administration of intravenous diphenhydramine. These cases demonstrate that swelling associated with influenza or its treatment can range from mild symptoms to life-threatening airway obstruction.

Influenza continues to cause significant illness worldwide, particularly in older adults and patients with multiple chronic conditions [1,2]. Because oseltamivir remains a first-line antiviral agent, clinicians should be aware that although hypersensitivity reactions are rare, they can occur. In patients receiving ACE inhibitors, distinguishing between medication-related angioedema and other potential causes may be difficult. A careful medication history and assessment of the temporal relationship between medication exposure and symptom onset are therefore important.

This case adds to the limited number of reports describing possible oseltamivir-associated angioedema. The close temporal relationship between oseltamivir administration and the onset of upper-lip swelling supports oseltamivir as a possible trigger; however, this association is heavily confounded by concurrent long-term lisinopril use, and a definitive causal relationship cannot be established. The absence of recurrent swelling during the available follow-up further supports clinical resolution, although the subsequent antihypertensive medication regimen was not available to the treating hospitalist team. This case therefore emphasizes the importance of considering multiple potential causes of facial swelling in patients with influenza. Early recognition, close airway monitoring, and prompt discontinuation of suspected medications are important to reduce the risk of serious complications.

## Conclusions

Oseltamivir-associated hypersensitivity reactions, although uncommon, remain clinically significant. In this case, the close temporal relationship between oseltamivir administration and the onset of upper-lip swelling suggests a possible oseltamivir-associated reaction; however, concurrent long-term lisinopril therapy represents an important confounding factor, and a definitive causal relationship cannot be established. In patients presenting with influenza infection, clinicians should remain vigilant for signs of angioedema, particularly when multiple potential triggers are present. Early recognition, discontinuation of suspected offending medications, and close airway monitoring are essential to reduce the risk of life-threatening complications.
